# Clinical Impact of Corrections to Infliximab and Adalimumab Monitoring Results with the Homogeneous Mobility Shift Assay

**DOI:** 10.3390/jcm9092840

**Published:** 2020-09-02

**Authors:** Konstantinos Papamichael, Valerio J. Thomas, Andrea Banty, William T. Clarke, Katharine A. Germansky, Sarah N. Flier, Joseph D. Feuerstein, Gil Y. Melmed, Adam S. Cheifetz

**Affiliations:** 1 Division of Gastroenterology, Beth Israel Deaconess Medical Center, Boston, MA 02215, USA; tvalerio@bidmc.harvard.edu (V.J.T.); wclarke@bidmc.harvard.edu (W.T.C.); kgermans@bidmc.harvard.edu (K.A.G.); sflier1@bidmc.harvard.edu (S.N.F.); jfeuerst@bidmc.harvard.edu (J.D.F.); acheifet@bidmc.harvard.edu (A.S.C.); 2 Division of Gastroenterology, Cedars-Sinai Medical Center, Los Angeles, CA 90048, USA; Andrea.Bandy@cshs.org (A.B.); gil.melmed@cshs.org (G.Y.M.)

**Keywords:** anti-TNF therapy, therapeutic drug monitoring, homogenous mobility shift assay, inflammatory bowel disease

## Abstract

An upward drift for both infliximab and adalimumab concentrations measured by the homogenous mobility shift assay (HMSA) was previously reported. We aimed to investigate the impact of this drift on clinical care of patients with inflammatory bowel disease. This was a retrospective, multicenter study. Providers reviewed the individual patient data and drug concentrations before and after the laboratory corrections and then documented whether a different clinical decision would have been made had the corrected drug concentration been originally reported. A multivariable Cox proportional hazards regression analysis was performed to investigate the association of a documented treatment change with treatment failure, defined as drug discontinuation for primary nonresponse, loss of response, or serious adverse event, adjusting for confounding factors. The study population consisted of 479 patients (infliximab, *n* = 219; adalimumab, *n* = 260). Upon review, 14.9% (71/479) patients would have had a different treatment decision made had the corrected drug concentration been initially reported. After a median follow-up of 10.6 months, 25.7% of patients (123/479) had treatment failure. A theoretical different clinical decision based on the corrected drug concentrations was not associated with treatment failure (adjusted hazard ratio (HR): 1.452; 95% confidence interval (CI): 0.805–2.618; *p* = 0.216), which was consistent for both infliximab (adjusted HR: 1.977; 95% CI: 0.695–5.627; *p* = 0.201) and adalimumab (adjusted HR: 1.484; 95% CI: 0.721–3.054; *p* = 0.284). The drift in infliximab and adalimumab concentrations in the HMSA assay affected treatment decisions in 15% of cases. However, this discrepancy was not associated with a higher cumulative probability for treatment failure.

## 1. Introduction

The use of therapeutic drug monitoring (TDM) of antitumor necrosis factor (anti-TNF) therapies has become more common for the treatment of inflammatory bowel disease (IBD). Reactive TDM has transformed the management of primary nonresponse (PNR) and loss of response (LOR) among anti-TNF-treated patients [1]. Additionally, evidence suggests that proactive TDM is associated with better therapeutic outcomes compared to empiric treatment optimization and/or reactive TDM in patients with IBD treated with either infliximab or adalimumab [2,3,4,5,6]. Utilization of TDM and consequently TDM-based therapeutic decisions in real-world clinical practice relies heavily on the thresholds used to define a therapeutic drug concentration. Based on these, physicians must decide, for example, to change therapy when a patient has active IBD despite therapeutic drug concentrations, increase drug when a patient has subtherapeutic concentrations, or de-escalate treatment when a patient is in remission and has supratherapeutic drug concentrations. However, these thresholds can vary depending on the targeted clinical outcome, IBD phenotype, and TDM assay used [7]. Regarding the latter, we have previously shown a discrepancy between a commercially available enzyme-linked immunosorbent assay (ELISA) and a commercially available homogeneous mobility shift assay (HMSA), for both infliximab and adalimumab concentrations [8,9]. Based on these results, a comprehensive review of the HMSA assays was initiated and an upward drift for both infliximab (from December 2017 to May 2019) and adalimumab (from August 2017 to May 2019) was found, including the period during which our study was performed. The errant values were then corrected and the revised drug concentrations were reported to physicians. The HMSA is a drug tolerant assay widely used in the United States.

The primary aim of this study was to investigate the impact of this assay drift for infliximab and adalimumab concentrations on the clinical care of patients with IBD. Physicians were queried whether a different clinical decision would have been made had the corrected drug concentration been originally reported. We then assessed the association of a retrospective (theoretical) treatment change with treatment failure. The secondary outcome was to evaluate the quantitative and qualitative differences of infliximab and adalimumab concentrations after the correction of the HMSA.

## 2. Experimental Section

This is a retrospective, cohort multicenter study reflecting real-world clinical practice of eleven physicians and two nurse practitioners at two tertiary IBD centers. Consecutive IBD patients treated with infliximab or adalimumab therapy who underwent TDM within the affected time window for each drug were included in the study. Providers reviewed the individual patient data and drug concentrations before and after the drug assay corrections. Based on the corrected concentrations, the providers documented whether they would have made a different clinical decision and what the new decision would have been. A retrospective (theoretical) treatment change was “treatment escalation” instead of “drug discontinuation”, “treatment escalation” instead of “no change”, or “no change” as opposed to “treatment de-escalation”. “Treatment escalation” included dose increase, interval shortening, and/or an addition of an immunomodulator (thiopurine or methotrexate), while “treatment de-escalation” included dose decrease, interval prolongation, and/or immunomodulator discontinuation if on combination therapy with anti-TNF and immunomodulator. Patients were followed from the date of the last affected drug concentration until treatment failure or the end of December 2019. Treatment failure was defined as the need for drug discontinuation due to PNR, LOR, and disease flare or a serious adverse event. Demographic and clinical characteristics of the patients were acquired via their electronic medical records. The study was approved by the institutional review boards of the Beth Israel Deaconess Medical Center and Cedars–Sinai Medical Center.

Descriptive statistics were provided with median and interquartile range (IQR) for continuous variables, and frequency and percentages for categorical variables. A survival analysis was performed from the last affected TDM within the affected time window until treatment failure or end of follow-up, with the primary predictor of whether there was a discordance between the actual therapeutic decision and the retrospective (theoretical) decision had the corrected value been known. Kaplan–Meier estimates were used to draw the cumulative incidence curves, compared by log-rank test. A multivariable Cox proportional hazards regression analysis was also performed to investigate the association of a retrospective (theoretical) treatment change with treatment failure, adjusting for potential confounding factors including gender, IBD type, number of affected samples per patient, TDM type (reactive vs. proactive), anti-TNF therapy (adalimumab vs. infliximab), IBD center, at least one TDM after last affected TDM, and at least one treatment optimization after last affected TDM. A receiver-operating characteristic (ROC) analysis was performed to identify drug concentration thresholds at last affected TDM associated with treatment failure either before or after the correction. Optimal thresholds were chosen using the Youden index. The last drug concentrations within the affected time window before and after the correction of the HMSA were also categorized into quartiles. Rates of treatment failure were compared across drug concentrations using the chi-square test (linear-by-linear association). Comparisons of drug concentrations before and after the correction of the HMSA (ANSER^®^, Prometheus Laboratories Inc. San Diego, CA, USA) were performed using the Wilcoxon-signed rank test. Qualitative agreement in drug concentration status between assays was expressed as the positive and negative percent agreement (correlating with therapeutic and subtherapeutic classification, respectively) using different cut-offs for therapeutic drug concentrations. The coefficient of agreement was reported using Cohen’s kappa (κ) and classified as almost perfect (>0.9), strong (0.8–0.9), moderate (0.6–0.79), weak (0.4–0.59), minimal (0.21–0.39), and none (0–0.2). Samples with no absolute values were excluded from the final analyses. All analyses were performed using SPSS version 25.0 (SPSS, Chicago, IL, USA) and GraphPad Prism version 5.03 for Windows (GraphPad Software, San Diego, CA, USA).

## 3. Results

### 3.1. Study Population

The study population consisted of 479 patients (CD, *n* = 361 (75.4%)) who were treated either with infliximab (*n* = 219, (45.7%)) or adalimumab (*n* = 260, (54.3%)) (Table 1). Patient demographic and clinical characteristics are shown in Table 1.

Approximately half of the patients were females and around three quarters of the patients had Crohn’s disease (CD). The majority of patients receiving infliximab underwent proactive TDM (81.3%), in contrast to 53.1% of the patients on adalimumab. One hundred forty-three (29.9%) patients had at least one TDM performed after the last affected sample (infliximab: 90/219 (41.1%) and adalimumab: 53/260 (20.4%)). Moreover, 86 out of the 479 patients (18%) had at least one treatment optimization after the last affected drug concentration (infliximab: 48/219 (21.9%) and adalimumab: 38/260 (14.6%)).

### 3.2. Retrospective (Theoretical) Treatment Change and Treatment Failure

Upon review, seventy-one (14.9%) patients would have had a different treatment decision made, had the corrected drug concentration been initially reported (infliximab, *n* = 44, 20.1%; adalimumab, *n* = 27, 10.4%) (Table 2).

After a median follow-up of 10.6 (IQR: 6.4–13.5) months, 123 (25.7%) patients had treatment failure (Table 3).

**Figure 1:**
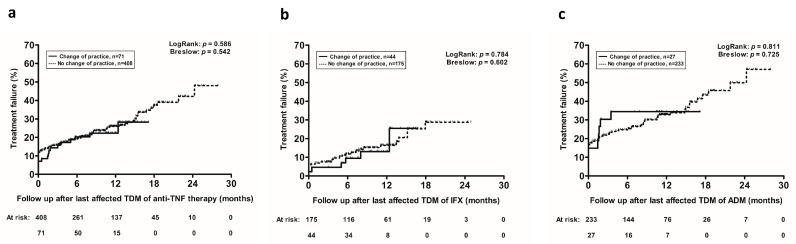
Kaplan–Meier cumulative probability curves of treatment failure in patients treated with (**a**) anti-TNF therapy with no treatment changes (dotted line) or a retrospectively (theoretically) different therapeutic decision (solid line) following correction of originally reported results, stratified also by anti-TNF therapy type, (**b**) infliximab, or (**c**) adalimumab. Abbreviations: IFX: infliximab; ADM: adalimumab; TDM: therapeutic drug monitoring; anti-TNF: anti-tumor necrosis factor.

Kaplan–Meier analysis demonstrated a comparable overall cumulative probability of treatment failure in patients with or without a retrospective (theoretical) treatment change (log-rank *p* = 0.586, Figure 1a), which was consistent for both infliximab (log-rank *p* = 0.784, Figure 1b) and adalimumab (log-rank *p* = 0.811, Figure 1c).

After adjusting for potential confounding factors, a retrospective (theoretical) treatment change was not associated with treatment failure (hazard ratio (HR): 1.452; 95%CI: 0.805–2.618; *p* = 0.216), which was consistent for both infliximab- (HR: 1.977; 95%CI: 0.695–5.627; *p* = 0.201) and adalimumab-treated patients (HR: 1.484; 95%CI: 0.721–3.054; *p* = 0.284).

### 3.3. Drug Concentrations and Treatment Failure

**Figure 2:**
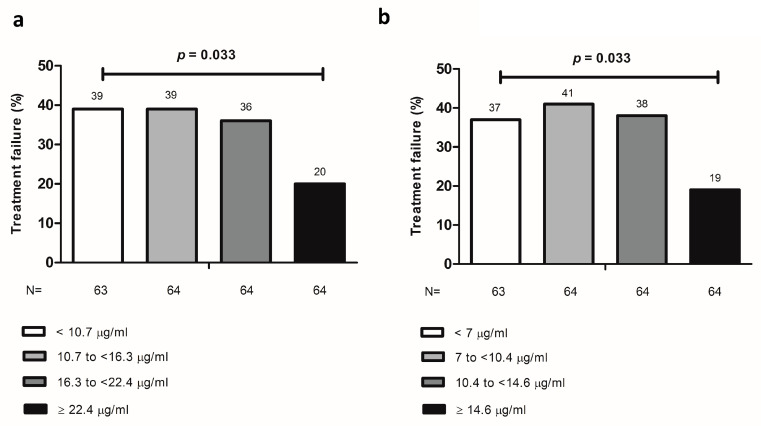
Treatment failure by quartiles of adalimumab concentration at last affected therapeutic drug monitoring before (**a**) or after (**b**) the implementation of the corrected measures.

Based on ROC analysis, we were not able to identify an infliximab drug concentration threshold either before or after the correction of the HMSA evaluated at the last drug concentration affected by the drift that discriminated patients with or without treatment failure (supplementary Figure S1A). However, an adalimumab concentration of 22.4 µg/mL (sensitivity: 30.6%, specificity: 85.9%) before the correction of the HMSA and 14.4 µg/mL (sensitivity: 31.2%, specificity: 85.9%) after the correction of the HMSA were identified as cutoffs discriminating patients with or without treatment failure; lower drug concentrations were associated with higher likelihood of treatment failure (supplementary Figure S1B). The relationship between adalimumab concentrations at the last drug concentration affected by the drift and treatment failure was further analyzed by dividing drug concentrations into quartiles. The Q1–Q3 adalimumab quartiles, both before and after the corrected HMSA, were associated with a significant 2-fold increased unadjusted rate of treatment failure compared to the Q4 quartile. However, the quartiles of adalimumab concentrations were numerically different before and after the correction of the HMSA (Figure 2).

### 3.4. Comparison of Drug Concentrations

**Figure 3:**
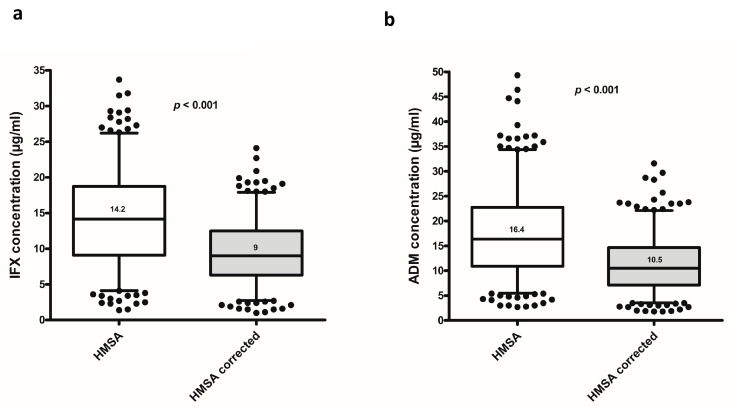
Drug concentrations regarding (**a**) infliximab and (**b**) adalimumab at the last affected TDM before and after the correction of the homogeneous mobility shift assay. Box whisker plots show the median (solid line within box), interquartile range (upper and lower box boundaries), and 5–95% lower and upper extreme (whiskers). Circles represent outliers. Abbreviations: IFX: infliximab; ADM: adalimumab; TDM: therapeutic drug monitoring.

For infliximab, 318 samples were analyzed. Following the correction of the HMSA infliximab concentrations (median (interquartile range, IQR)) were significantly lower compared to those before the correction of the HMSA (9 (6.3–12.5) μg/mL vs. 14.2 (9.1–18.8) μg/mL; *p* < 0.001, respectively, *n* = 284, Figure 3a). Qualitative agreement in infliximab concentration status (therapeutic vs. subtherapeutic) before and after the correction of the HMSA was only weak using >5 μg/mL (*κ* = 0.516, *p* < 0.001), >7 μg/mL (*κ* = 0.577, *p* < 0.001), or >10 μg/mL (*κ* = 0.514, *p* < 0.001) as therapeutic drug concentrations. Qualitative agreement between infliximab concentrations before and after the correction of the HMSA based on different cut-offs for therapeutic drug concentrations is described in supplementary Table S1.

For adalimumab, 338 samples were analyzed. Following the correction of the HMSA adalimumab concentrations (median (IQR)) were significantly lower compared to those before the correction of the HMSA (10.5 (7.1–14.7) μg/mL vs. 16.4 (10.9–22.8) μg/mL; *p* < 0.001, respectively, *n* = 328, Figure 2b). Qualitative agreement in adalimumab concentration status (therapeutic vs. subtherapeutic) before and after the correction of the HMSA was only weak using >5 μg/mL κ = 0.571, *p* < 0.001), >7 μg/mL (κ = 0.541, *p* < 0.001), or >10 μg/mL (κ = 0.490, *p* < 0.001) as therapeutic drug concentrations. Qualitative agreement between drug concentrations before and after the correction of the HMSA based on different cut-offs for therapeutic drug concentrations is described in supplementary Table S1.

## 4. Discussion

Both reactive and proactive TDM are being used more frequently in everyday clinical practice as a tool to better optimize anti-TNF therapy in IBD and achieve favorable therapeutic outcomes [10,11,12]. Several assays are now available for measuring drug concentrations and antidrug antibodies to anti-TNF therapies [13]. The ELISA and the HMSA are the most commonly used tools to measure drug concentrations and antidrug antibodies. An upward drift for both infliximab and adalimumab concentrations measured by the HMSA was previously found, followed by a correction of the values that was then reported to the treatment providers. In our population of 479 patients, 15% would have had a different clinical decision made if the corrected value was originally reported. However, the overall cumulative probability for treatment failure was comparable for both infliximab and adalimumab, irrespective of whether a treatment decision might have been different had the corrected result been originally reported. Overall, treatment failure was higher in patients on adalimumab than on infliximab therapy, but this may be explained by the fact that fewer adalimumab-treated patients had at least one drug concentration measured (20.4% vs. 41.1%) and treatment optimization (14.6% vs. 21.9%) after the last sample affected by the drift compared to infliximab-treated patients. Thus, continued TDM eventually led to the reporting of the correct drug concentration and eventual dose optimization. Additionally, less adalimumab- than infliximab-treated patients underwent at least one proactive TDM during the affected time period (56.2% vs. 84.4%, respectively).

We also found that infliximab and adalimumab concentrations before and after the correction of the HMSA were significantly different, both quantitatively and qualitatively. This is in line with previous studies showing discrepancies among assays used for evaluating biologic drug concentrations [8,9,14,15]. Moreover, we identified adalimumab concentration thresholds of 22.4 µg/mL before the corrected measures and 14.4 µg/mL after the corrected measures discriminated patients with or without treatment failure. This is the first study to show that assay-related differences in drug concentrations can directly influence corresponding drug concentration thresholds associated with clinical outcomes. This is important as physicians often base their decisions on targeting certain drug concentration thresholds. For this reason, commercial assays should be accurately cross-validated and standardized, as significant differences in drug concentrations can lead to diverse therapeutic decisions that may significantly impact clinical outcomes.

Strengths of the study are the large sample size and the fact that it represents the real-world clinical practice in two large tertiary IBD centers. However, limitations of the study are its retrospective design and the fact that the impact of corrections to infliximab and adalimumab monitoring results on objective therapeutic outcomes, such as mucosal healing, was not evaluated.

In conclusion, we found that the HMSA infliximab and adalimumab assay drift led to providers reporting a change in care of 15% of patients based on the corrected value. However, these decisions did not lead to a higher rate of treatment failure compared to patients without a retrospective (theoretical) treatment change. There were also significant quantitative and qualitative differences between infliximab and adalimumab concentrations before and after the correction of the HMSA, directly influencing drug concentration thresholds associated with clinical outcomes.

## Figures and Tables

**Table 1:** Patients’ demographic and clinical characteristics.

Patients’ Characteristics	Infliximab (*n* = 219)	Adalimumab (*n* = 260)	Total (*n* = 479)
Gender: Female, (%)	108 (49.3)	132 (50.8)	240 (50.1)
IBD type (%)			
CD	143 (65.3)	218 (83.8)	361 (75.4)
UC	72 (32.9)	36 (13.8)	108 (22.5)
UIBD	4 (1.8)	6 (2.4)	10 (2.1)
Number of affected samples per patient, median, range	1 (1–6)	1 (1–4)	1 (1–6)
TDM type (%)			
Proactive	178 (81.3)	138 (53.1)	316 (66)
Reactive	34 (15.6)	114 (43.8)	148 (30.9)
Both reactive and proactive	7 (3.1)	8 (3.1)	15 (3.1)
BIDMC, *n* (%)	117 (53.4)	93 (35.8)	210 (43.8)

CD: Crohn’s disease; UC: ulcerative colitis; BIDMC: Beth Israel Deaconess Medical Center; UIBD: undifferentiated inflammatory bowel disease; IQR: interquartile range; TDM: therapeutic drug monitoring; IBD: inflammatory bowel disease.

**Table 2:** A per patient analysis of therapeutic discrepancies noted after reporting of corrected results.

Original Therapeutic Decision Based on Originally Reported Results *	Retrospectively Determined (Theoretical) Therapeutic Decision after the Correction of the HMSA Results *	*N* (%)
Infliximab (*n* = 219)		
No change	Treatment escalation	27 (12.3)
Treatment de-escalation	No change	13 (5.9)
Drug discontinuation	Treatment escalation	2 (0.9)
Both no change and treatment de-escalation *	Both treatment escalation and no change	2 (0.9)
		Total 44 (20.1)
Adalimumab (*n* = 260)		
No change	Treatment escalation	14 (5.4)
Treatment de-escalation	No change	7 (2.7)
Drug discontinuation	Treatment escalation	6 (2.3)
Both no change and treatment de-escalation	Both treatment escalation and no change	0 (0)
		Total 27 (10.4)
Total (*n* = 479)		
No change	Treatment escalation	41 (8.6)
Treatment de-escalation	No change	20 (4.2)
Drug discontinuation	Treatment escalation	8 (1.7)
Both no change and treatment de-escalation	Both treatment escalation and no change	2 (0.4)
		Total 71 (14.9)

* Some patients had multiple TDM during the study period. Abbreviations: HMSA: homogeneous mobility shift assay.

**Table 3:** Treatment failure per retrospectively determined (theoretical) therapeutic decision status.

Retrospective (Theoretical) Treatment Change Status	Infliximab	Adalimumab	Total
Patients with a retrospective (theoretical) treatment change			
Overall treatment failure, (%)	6 (13.6)	9 (33.3)	15 (21.1) ^a^
PNR	0	0	0
LOR	6 (13.6)	9 (33.3)	15 (21.1)
SAE	0	0	0
Patients without a retrospective (theoretical) treatment change			
Overall treatment failure, (%)	29 (16.6)	79 (33.9)	108 (26.5)
PNR	1 (0.6)	2 (0.8)	3 (0.7)
LOR	20 (11.4)	71 (30.5)	91 (22.3)
SAE	8 (4.6)	6 (2.5)	14 (3.5)
Total cohort			
Overall treatment failure, (%)	35 (16)	88 (33.8)	123 (25.7)
PNR	1 (0.5)	2 (0.7)	3 (0.7)
LOR	26 (11.9)	80 (30.8)	106 (22.1)
SAE	8 (3.6)	6 (2.3)	14 (2.9)

^a^ no change to treatment escalation, *n* = 7; drug discontinuation to treatment escalation, *n* = 7, treatment de-escalation to no change, *n* = 1. Abbreviations: TNF: tumor necrosis factor; PNR: primary non-response; LOR: loss of response; SAE: serious adverse event.

## Supplementary Materials

The following are available online at https://www.mdpi.com/2077-0383/9/9/2840/s1.
