# Supplementary material for: Clinical Impact of Corrections to Infliximab and Adalimumab Monitoring Results with the Homogeneous Mobility Shift Assay
Source: J Clin Med. 2020 Sep 2;9(9):2840. doi: 10.3390/jcm9092840 (PMC7565738; doi:10.3390/jcm9092840)
Supplement: Supplementary file 1 [file jcm-09-02840-s001.pdf]

Supplementary Table S1. Qualitative agreement in infliximab and adalimumab concentrations before and after the correction of the homogeneous mobility shift assay.

**INFLIXIMAB**					
		**HMSA corrected**			
>5 µg/ml		Sub-therapeutic	Therapeutic	Total	Percentage agreement
**HMSA**	Sub-therapeutic	22	0	22	Negative agreement = 39%
	Therapeutic	34	262	296	Positive agreement = 100%
	Total	56	262	318	Total agreement = 89%
		**HMSA corrected**			
>7 µg/ml		Sub-therapeutic	Therapeutic	Total	Percentage agreement
**HMSA**	Sub-therapeutic	44	1	45	Negative agreement = 49%
	Therapeutic	45	228	273	Positive agreement = 100%
	Total	89	229	318	Total agreement = 86%
		**HMSA corrected**			
>10 µg/ml		Sub-therapeutic	Therapeutic	Total	Percentage agreement
**HMSA**	Sub-therapeutic	84	0	84	Negative agreement = 52%
	Therapeutic	78	156	234	Positive agreement = 100%
	Total	162	156	318	Total agreement = 75%
**ADALIMUMAB**					
		**HMSA corrected**			
>5 µg/ml		Sub-therapeutic	Therapeutic	Total	Percentage agreement
**HMSA**	Sub-therapeutic	20	0	20	Negative agreement = 43%
	Therapeutic	26	292	318	Positive agreement = 100%
	Total	46	292	338	Total agreement = 92%
		**HMSA corrected**			
>7 µg/ml		Sub-therapeutic	Therapeutic	Total	Percentage agreement
**HMSA**	Sub-therapeutic	39	0	39	Negative agreement = 44%
	Therapeutic	49	250	299	Positive agreement = 100%
	Total	88	250	338	Total agreement = 86%
		**HMSA corrected**			
>10 µg/ml		Sub-therapeutic	Therapeutic	Total	Percentage agreement
**HMSA**	Sub-therapeutic	77	0	77	Negative agreement = 48%
	Therapeutic	84	177	261	Positive agreement = 100%
	Total	161	177	338	Total agreement = 75%

HMSA: homogeneous mobility shift assay.

Supplementary Figure S1. Receiver operator curve analysis for adalimumab concentrations at last affected TDM before (A) and after (B) the implementation of corrected measures stratifying patients with or without treatment failure.

A B

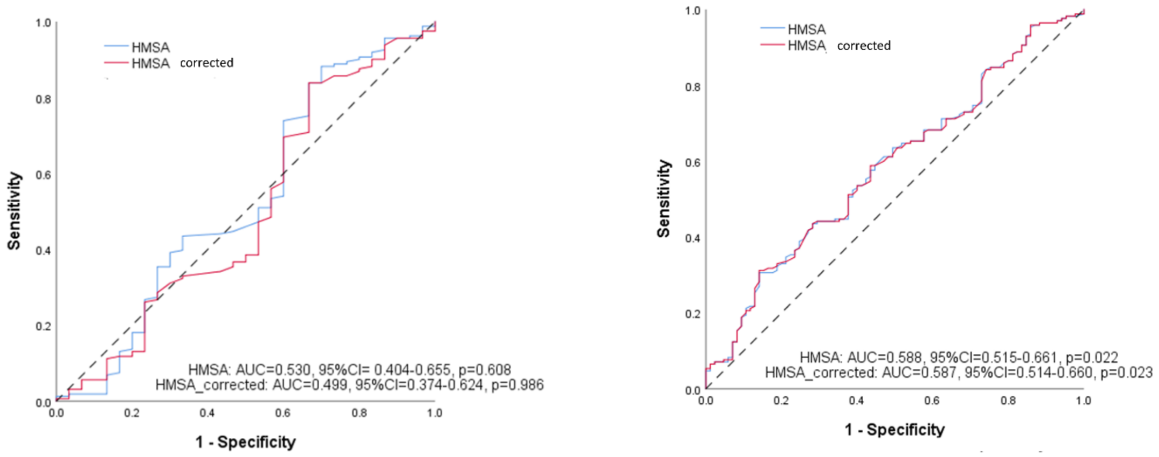

HMSA: homogeneous mobility shift assay; AUC: area under the curve; CI: confidence intervals.
